# Supplementary material for: Molecular basis for the phosphorylation of bacterial tyrosine kinase Wzc
Source: Nat Commun. 2025 Apr 11;16:3437. doi: 10.1038/s41467-025-58693-7 (PMC11986026; doi:10.1038/s41467-025-58693-7)
Supplement: Supplementary file 1 — Supplementary Information [file 41467_2025_58693_MOESM1_ESM.pdf]

Supplementary Table 1. Cryo-EM data collection, refinement and validation statistics.

	Wzc^{K540M}2YE ADP complex		Wzc^{K540M}3YE ADP complex		Wzc^{K540M}3YE_N711Y ADP complex	
	(EMD-50042) (PDB 9EXO)	(EMD-50043) (PDB 9EXP)	(EMD-50044) (PDB 9I2Q)	(EMD-50045) (PDB 9I2R)	(EMD-50046) (PDB 9EXQ)	(EMD-50047) (PDB 9EXR)
**Data collection and processing**						
Magnification	165,000	165,000	165,000	165,000	165,000	165,000
Voltage (kV)	300	300	300	300	300	300
Electron exposure (e–/Å^2)	40.3; 37.3; 37.5	40.3; 37.3; 37.5	38.3; 37.8	38.3; 37.8	31.9	31.9
Defocus range (μm)	-0.8~-2.0	-0.8~-2.0	-0.8~-2.0	-0.8~-2.0	-0.8~-2.4	-0.8~-2.4
Pixel size (Å)	0.737	0.737	0.737	0.737	0.737	0.737
Symmetry imposed	C1	C8	C1	C8	C1	C8
Final particle images (no.)	528,899	528,899	519,340	519,340	413,751	413,751
Map resolution (Å)	3.00	2.85	2.90	2.59	2.71	2.49
FSC threshold	0.143	0.143	0.143	0.143	0.143	0.143
Map sharpening *B* factor (Å^2)	-98.1	-128.8	-80.8	-96.0	-88.9	-104.3
**Refinement**						
Model resolution (Å)	3.2	3.0	3.2	2.9	2.9	2.5
FSC threshold	0.5	0.5	0.5	0.5	0.5	0.5
Model composition						
Non-hydrogen atoms	36,815	32,064	35,911	31,160	36,703	31,952
Protein residues	4,729	4,136	4,633	4,040	4,713	4,120
Ligands	16 (8 ADP, 8 Mg^{2+})	16 (8 ADP, 8 Mg^{2+})	16 (8 ADP, 8 Mg^{2+})	16 (8 ADP, 8 Mg^{2+})	16 (8 ADP, 8 Mg^{2+})	16 (8 ADP, 8 Mg^{2+})
B factors (Å^2)						
Protein	106.8	64.62	131.34	75.39	50.03	57.45
Ligand	59.08	30.17	62.27	34.31	28.10	8.4
R.m.s.d.						
Bond lengths (Å)	0.005	0.004	0.004	0.004	0.008	0.008
Bond angles (°)	0.630	0.558	0.566	0.560	0.716	0.788
Validation						
MolProbity score	1.66	1.57	1.71	1.62	1.75	1.82
Clashscore	6.67	5.66	7.20	6.13	8.41	10.31
Poor rotamers (%)	0	0	0	0	0	0
Ramachandran plot						
Favored (%)	95.84	96.17	95.43	95.90	95.78	95.86
Allowed (%)	4.16	3.83	4.57	4.10	4.22	4.14
Disallowed (%)	0	0	0	0	0	0

Supplementary Table 2. Summary of the MD simulation experiments. All systems were solvated with TIP3P waters and 0.15 M NaCl was added to neutralise the charge of the systems. Three replicates of 500 ns of MD simulation are for each system.

Kinase domain dimer					
	**Name**	**Modification**	**ADP**	**Mg^{2+}**	**# WATER**
**State 1**	WT	-	NO	NO	65968
	pY717 (1pY)	pY717	NO	NO	65978
	pY717_ADP	pY717	YES	NO	66020
	pY717_ADP_MG	pY717	YES	YES	65948
	1YE	Y717E	NO	NO	65957
**State 2**	WT	-	NO	NO	66012
	pY715	pY715	NO	NO	66007
	2pY7	pY717, pY715	NO	NO	66026
	2pY_ADP	pY717, pY715	YES	NO	65987
	2pY_ADP_MG	pY717, pY715	YES	YES	66030
	2YE	Y717E, Y715E	NO	NO	66001
**State 3**	WT	-	NO	NO	65942
	pY713	pY713	NO	NO	65937
	3pY	pY717, pY715, pY713	NO	NO	65973
	3pY_ADP	pY717, pY715, pY713	YES	NO	65978
	3pY_ADP_MG	pY717, pY715, pY713	YES	YES	65980
	3YE	Y717E, Y715E, Y717E	NO	NO	65944
**State 4**	WT	-	NO	NO	65920
	pY708	pY708	NO	NO	65958
	4pY	pY717, pY715, pY713, pY708	NO	NO	66003
	4pY_ADP	pY717, pY715, pY713, pY708	YES	NO	65947
	4pY_ADP_MG	pY717, pY715, pY713, pY708	YES	YES	65944
	4YE	Y717E, Y715E, Y713E, Y708E	NO	NO	65969

Dimer						
**Name**	**Modification**	**ADP**	**Mg^{2+}**	**# WATER**	**#POPE**	**#POPG**
**State 1 – WT**	-	NO	NO	72390	464	116
**State 4 – 4pY**	pY717, pY715, pY713, pY708	NO	NO	78428	500	125
**State 4 – 4pY_ADP**	pY717, pY715, pY713, pY708	YES	NO	77981	500	125
**State 4 – 4YE**	Y717E, Y715E, Y713E, Y708E	YES	NO	78341	126	504
**Trimer**						
**Name**	**Modification**	**ADP**	**Mg^{2+}**	**# WATER**	**#POPE**	**#POPG**
**State 1 – WT**	-	NO	NO	101465	652	163
**State 4 – 4pY_ADP**	pY717, pY715, pY713, pY708	YES	NO	101897	652	163
**State 4 – 4YE**	Y717E, Y715E, Y713E, Y708E	YES	NO	101894	652	163

Supplementary Table 3. List of primers.

Primer Name	Sequence	Description
primer1	CTAGTCCAAGCGCAGGTATGACTTTTATTAGCAGTAA	To generate Wzc K540M mutation
primer2	TTACTGCTAATAAAAGTCATACCTGCGCTTGGACTAG	
primer3	TATGGCGAGTCTGAGGAAGATAAAAAACACCACCACCACCACCACTGACTGCAG	To generate Wzc Y715E/ Y717E/Y718E mutation
primer4	TTCCTCAGACTCGCCATAATGATTATGGCCATATCTATAGTAGCTGCT	
primer5	ACTATAGATATGGCCATTATCATTATGGCTATTCTTA	To generate Wzc N711Y mutation
primer6	TAAGAATAGCCATAATGATAATGGCCATATCTATAGT	
primer7	ACTATAGATATGGCCATTATCATTATGGCGAGTCTGA	To generate Wzc Y715E/ Y717E/Y718E/N711Y mutation on the basis of Wzc Y715E/ Y717E/Y718E
primer8	TCAGACTCGCCATAATGATAATGGCCATATCTATAGT	
primer9	TAATCATTATGGCTATTCTGAGGAAGATAAAAAACACCACCACC	To generate Wzc Y717E/Y718E mutation
primer10	GGTGGTGGTGTTTTTTATCTTCCTCAGAATAGCCATAATGATTA	

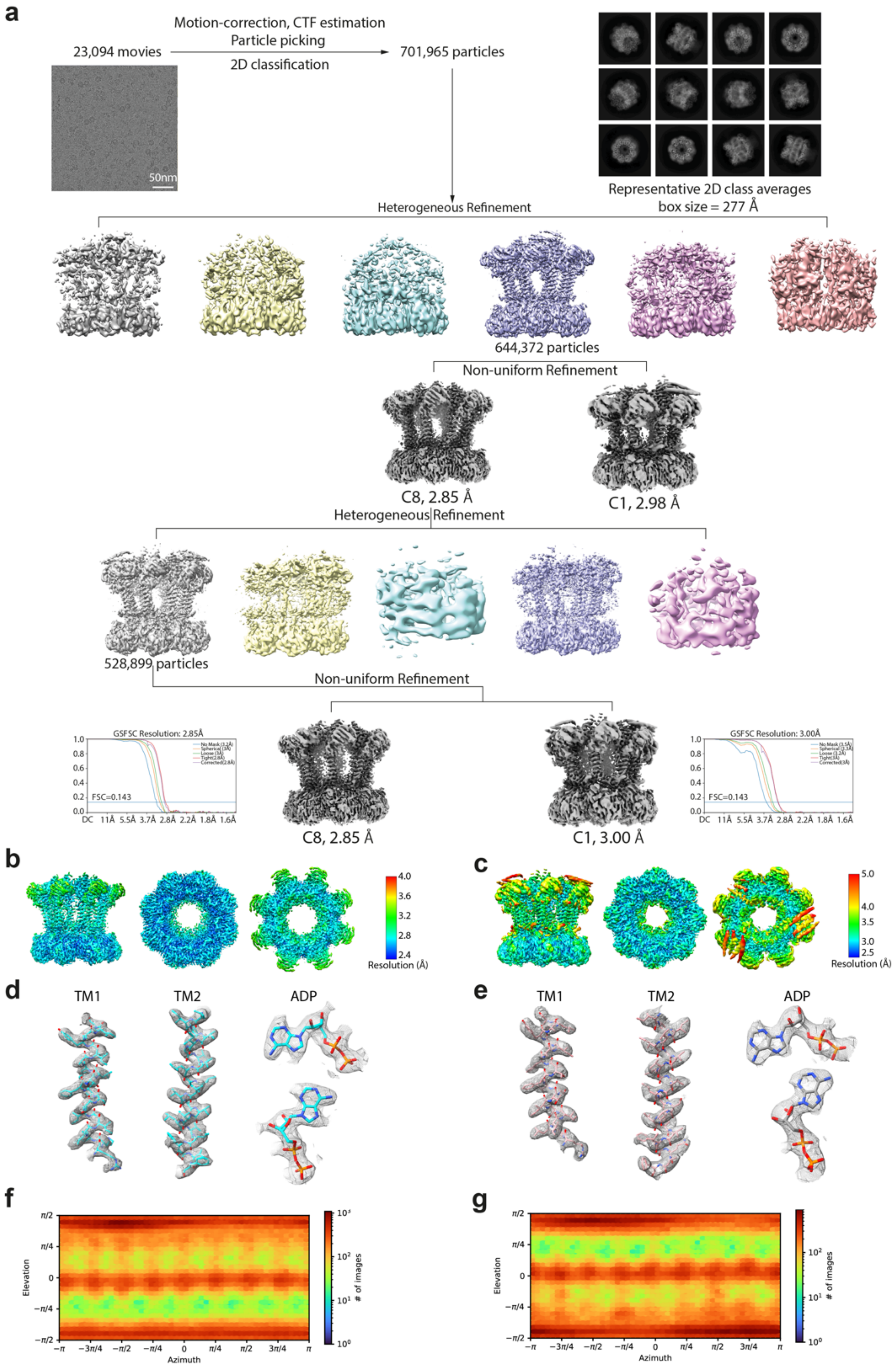

a
Motion-correction, CTF estimation
Particle picking
2D classification
23,094 movies
701,965 particles
50nm
Representative 2D class averages
box size = 277 Å
Heterogeneous Refinement
644,372 particles
Non-uniform Refinement
C8, 2.85 Å
C1, 2.98 Å
Heterogeneous Refinement
528,899 particles
Non-uniform Refinement
GSFSC Resolution: 2.85Å
FSC=0.143
C8, 2.85 Å
C1, 3.00 Å
GSFSC Resolution: 3.00Å
FSC=0.143
b
Resolution (Å)
c
Resolution (Å)
d
TM1
TM2
ADP
e
TM1
TM2
ADP
f
Elevation
Azimuth
# of images
g
Elevation
Azimuth
# of images

Supplementary Figure 1. Cryo-EM workflow of Wzc^{K540M}2YE in complex with ADP. a Overview of cryo-EM data collection and image processing workflow for Wzc^{K540M}2YE in complex with ADP Mg^{2+}. The FSC curves generated by cryoSPARC are shown. **b** Local resolution estimation for Wzc^{K540M}2YE in complex with ADP Mg^{2+} with C8 symmetry. **c** Local resolution estimation for Wzc^{K540M}2YE in complex with ADP Mg^{2+} with C1 symmetry. **d-e** Cryo-EM densities of C8 (**d**) and C1 (**e**) maps for representative regions: TM1 (transmembrane helix 1), TM2 (transmembrane helix 2) and ADP (two views). **f-g** Angular distribution plot of C8 (**f**) and C1 (**g**) particle sets.

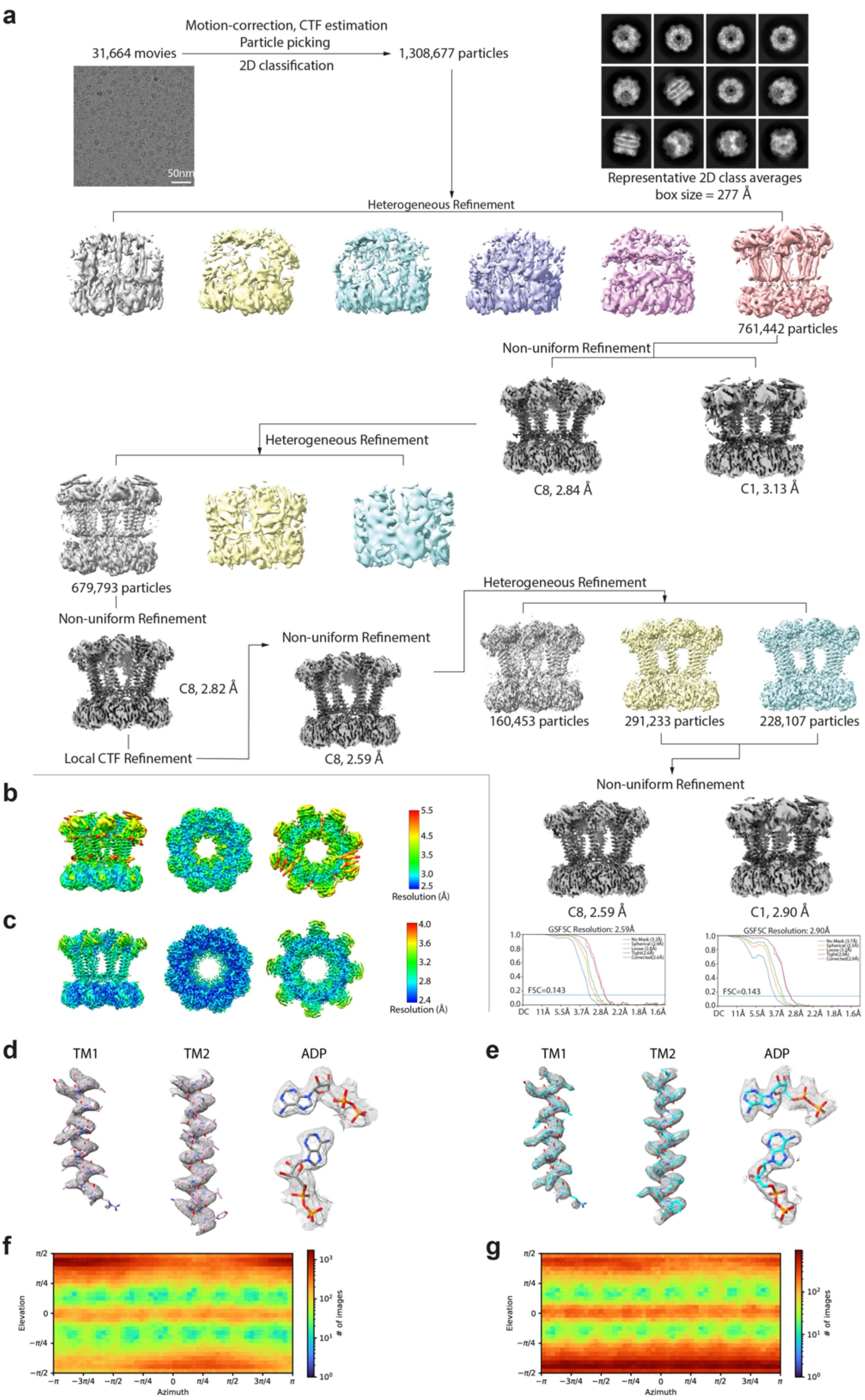
a
31,664 movies
Motion-correction, CTF estimation
Particle picking
2D classification
1,308,677 particles
50nm
Representative 2D class averages
box size = 277 Å
Heterogeneous Refinement
761,442 particles
Non-uniform Refinement
C8, 2.84 Å
C1, 3.13 Å
Heterogeneous Refinement
679,793 particles
Non-uniform Refinement
C8, 2.82 Å
Local CTF Refinement
Non-uniform Refinement
C8, 2.59 Å
Heterogeneous Refinement
160,453 particles
291,233 particles
228,107 particles
Non-uniform Refinement
C8, 2.59 Å
C1, 2.90 Å
GSFSC Resolution: 2.59Å
GSFSC Resolution: 2.90Å
FSC=0.143
DC 11Å 5.5Å 3.7Å 2.8Å 2.2Å 1.8Å 1.6Å
b
5.5
4.5
3.5
3.0
2.5
Resolution (Å)
c
4.0
3.6
3.2
2.8
2.4
Resolution (Å)
d
TM1
TM2
ADP
e
TM1
TM2
ADP
f
Elevation
Azimuth
# of images
g
Elevation
Azimuth
# of images

Supplementary Figure 2. Cryo-EM workflow of Wzc^{K540M}3YE in complex with ADP. a Overview of cryo-EM data collection and image processing workflow for Wzc^{K540M}3YE in complex with ADP Mg^{2+}. The FSC curves generated by cryoSPARC are shown. **b** Local resolution estimation for Wzc^{K540M}3YE in complex with ADP Mg^{2+} with C1 symmetry. **c** Local resolution estimation for Wzc^{K540M}3YE in complex with ADP Mg^{2+} with C8 symmetry. **d-e** Cryo-EM densities of C1 (**d**) and C8 (**e**) maps for representative regions: TM1 (transmembrane helix 1), TM2 (transmembrane helix 2) and ADP (two views). **f-g** Angular distribution plot of C1 (**f**) and C8 (**g**) particle sets.

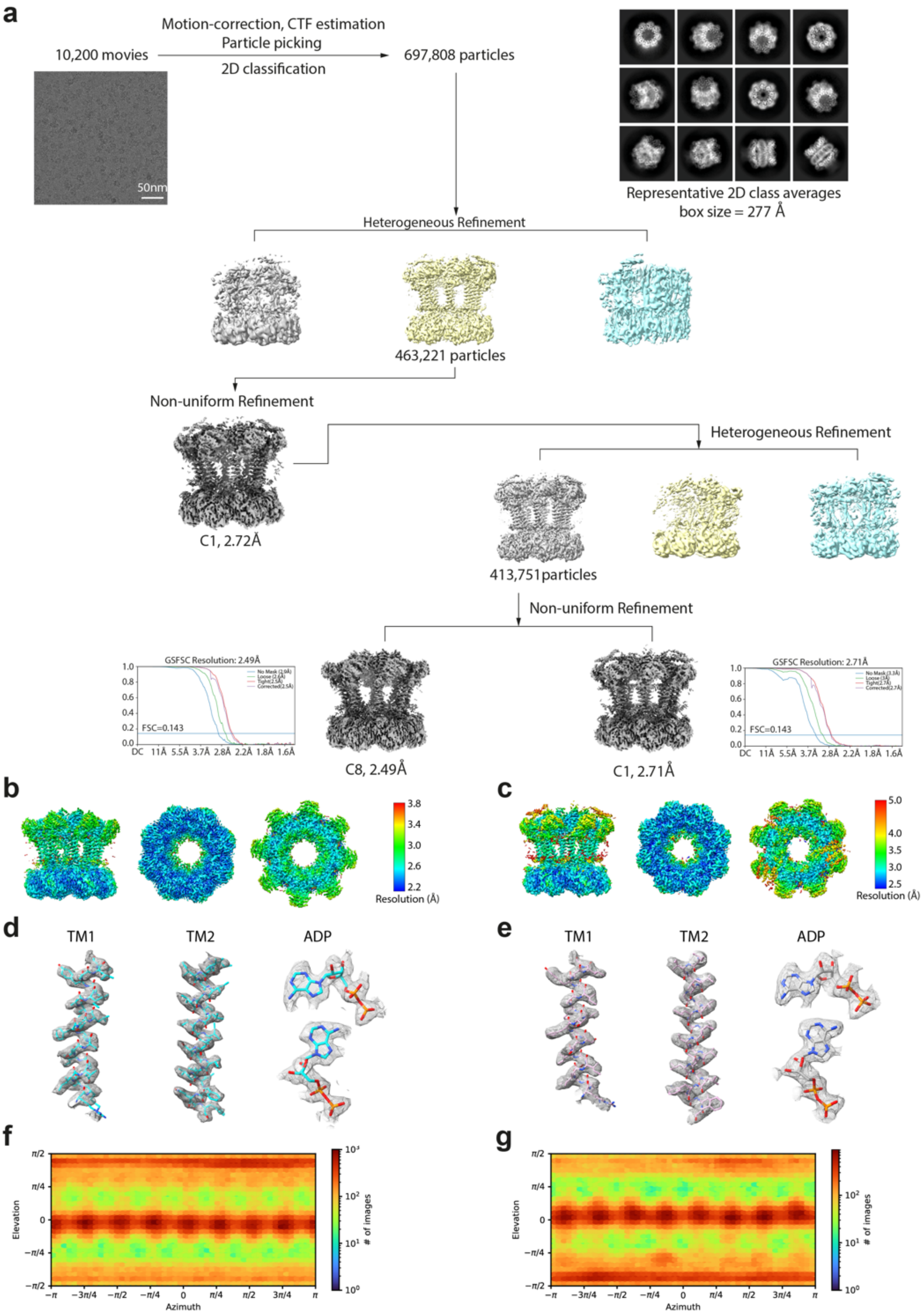

Supplementary Figure 3. Cryo-EM workflow of Wzc^{K540M}3YE_N711Y in complex with ADP. a Overview of cryo-EM data collection and image processing workflow for Wzc^{K540M}3YE_N711Y in complex with ADP Mg^{2+}. The FSC curves generated by cryoSPARC

are shown. **b** Local resolution estimation for Wzc^{K540M}3YE_N711Y in complex with ADP Mg^{2+} with C8 symmetry. **c** Local resolution estimation for Wzc^{K540M}3YE_N711Y in complex with ADP Mg^{2+} with C1 symmetry. **d-e** Cryo-EM densities of C8 (**d**) and C1 (**e**) maps for representative regions: TM1 (transmembrane helix 1), TM2 (transmembrane helix 2) and ADP (two views). **f-g** Angular distribution plot of C8 (**f**) and C1 (**g**) particle sets.

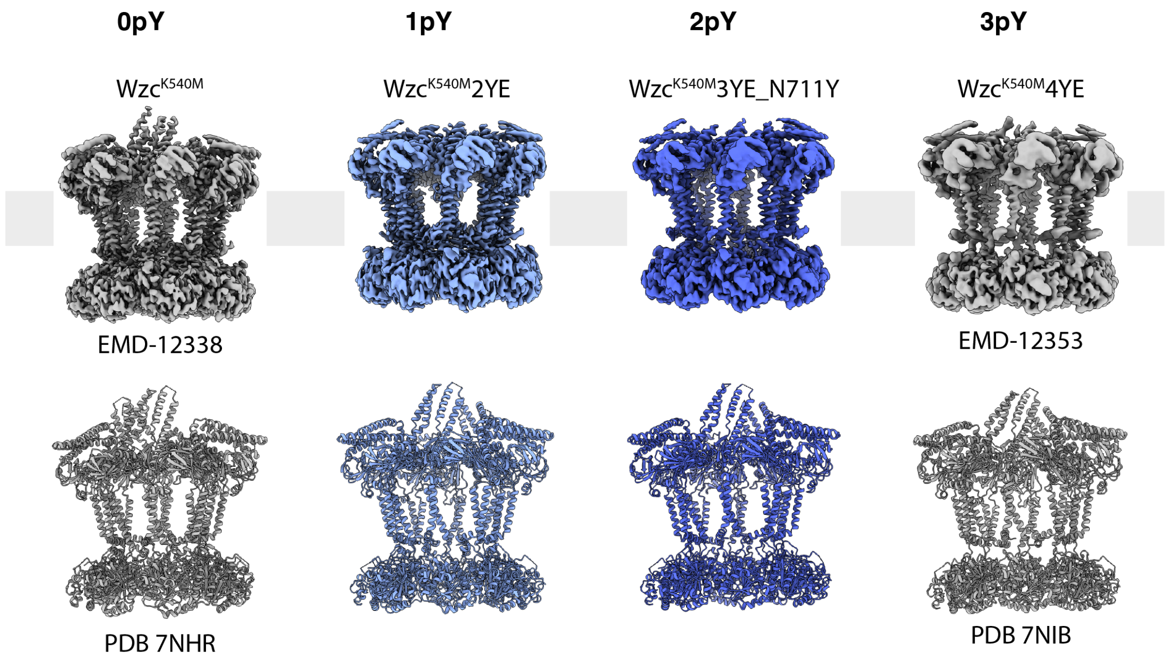

Supplementary Figure 4. Cryo-EM maps (top) and structures (bottom) of Wzc mutants representing different phosphorylation states.

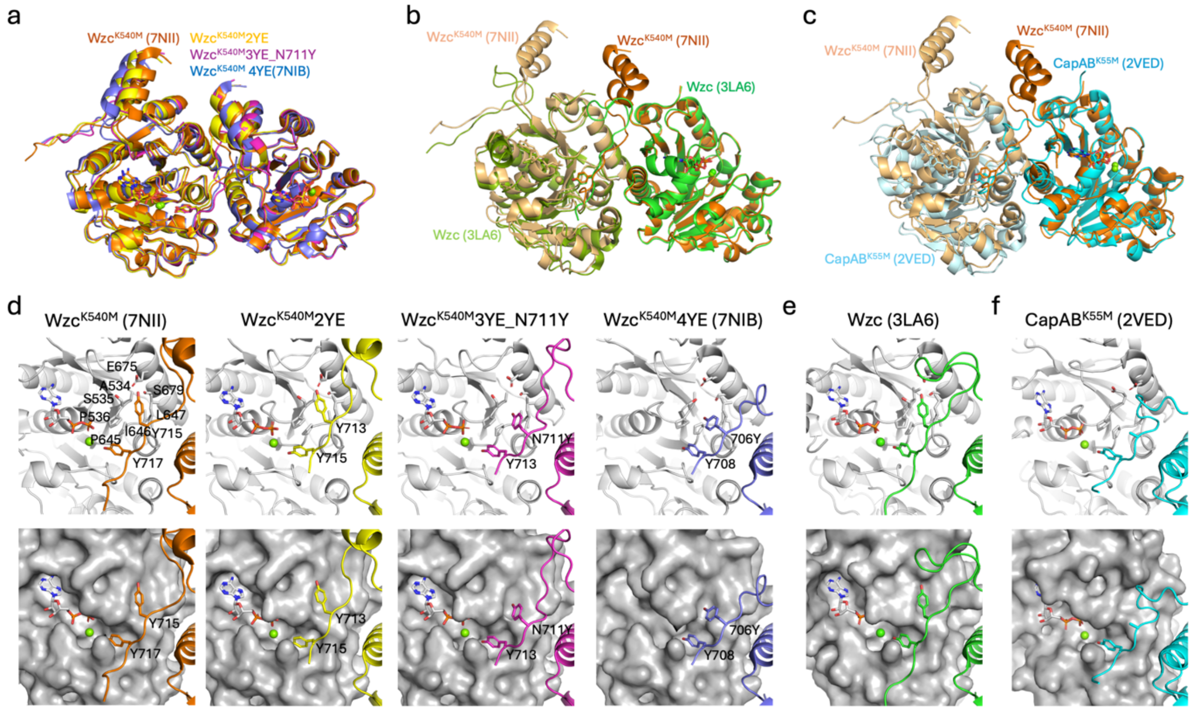

Supplementary Figure 5. Structural comparisons of the kinase domains. a Structure comparison of two adjacent kinase domains from full length Wzc from *E. coli* K30 in four different phosphorylation states. **b** Two kinase domains from full length Wzc^{K540M} (PDB ID: 7NII) superimposed with two kinase only domains of Wzc from *E. coli* K-12 (PDB ID: 3LA6) **c** Two kinase domain from Wzc^{K540M} (PDB ID: 7NII) superimposed with CapAB^{K55M} (PDB ID: 2VED). The monomer on the right was used for alignment. **d - f** View of the active site and the Tyr pocket. In each panel, the monomer providing the active site and the Tyr pocket is coloured gray, and the monomer providing the tyrosine tail is coloured. The upper structures show the essential residues contributing to the Tyr pocket, and the lower structures show the corresponding surface views. The key residues constituting the Tyr pocket are shown as sticks.

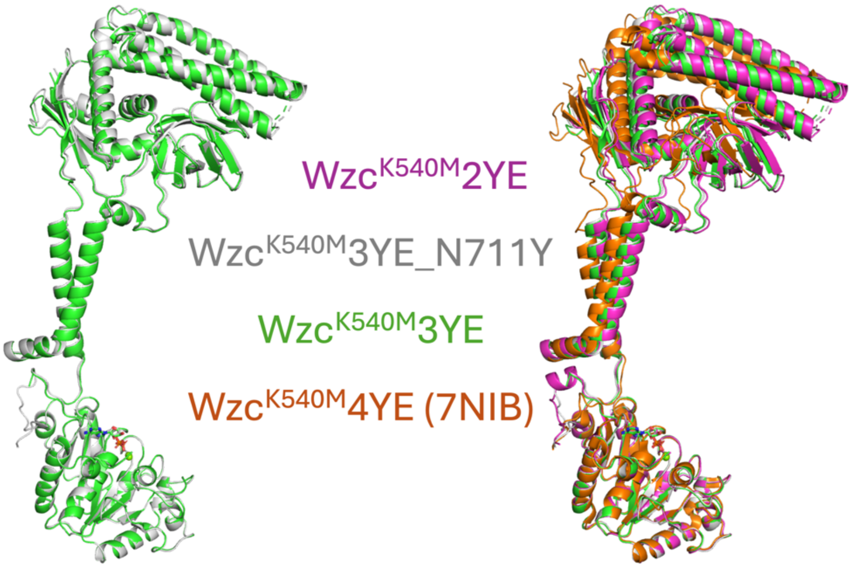

Supplementary Figure 6. Structural comparisons of Wzc monomers. The kinase domains are aligned. The structures of Wzc^{K540M}3YE_N711Y and Wzc^{K540M}3YE are very similar, with minor tilting of the transmembrane and periplasmic region (left). The conformations of the transmembrane and periplasmic region of both Wzc^{K540M}3YE and Wzc^{K540M}3YE_N711Y are in the middle of the conformations of Wzc^{K540M}2YE and Wzc^{K540M}4YE (right).

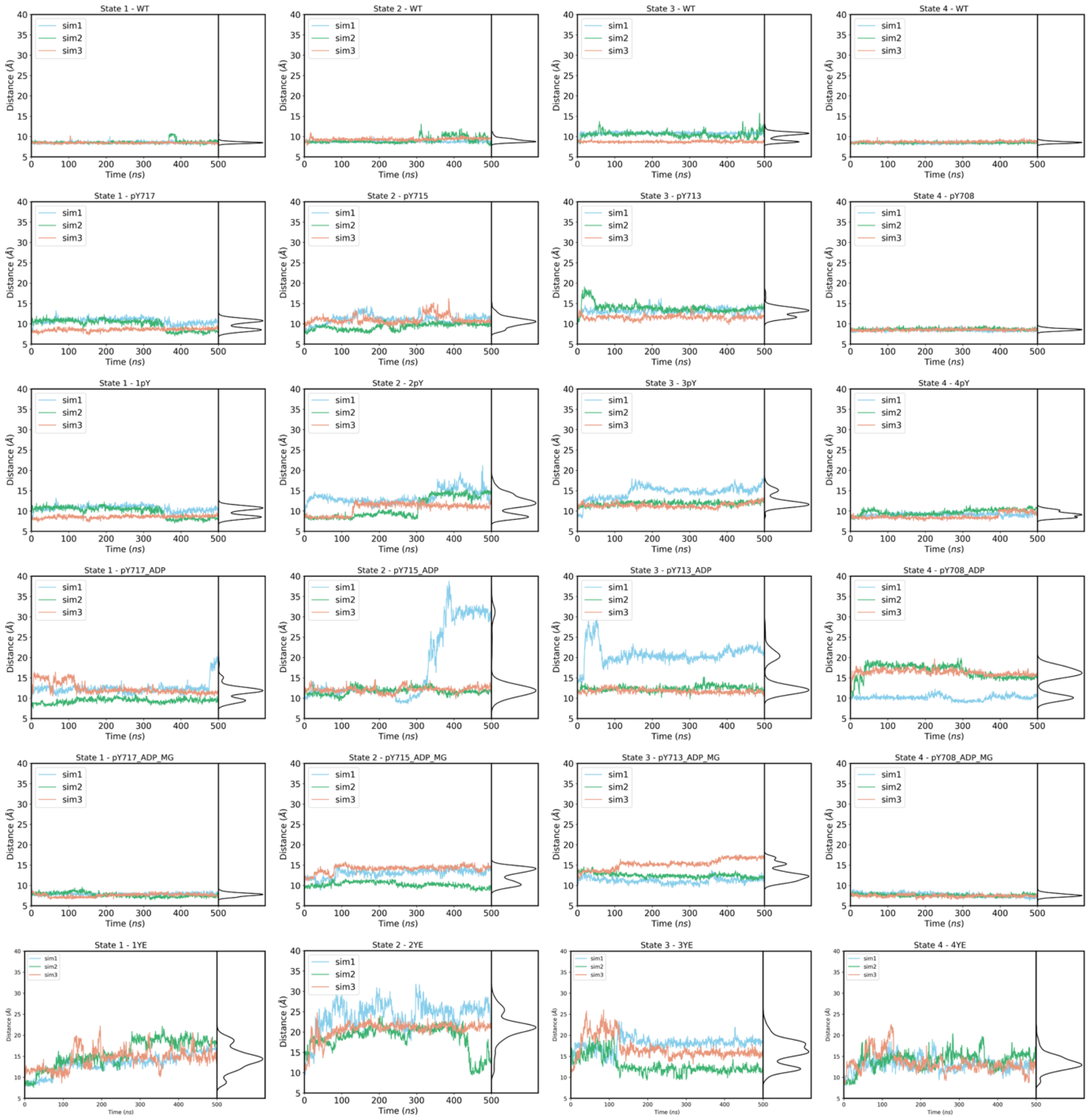

Supplementary Figure 7. Time traces of the measured distances between the Cβ of the tyrosine (or glutamic acid) and the Cα of D564 (located in the active site of the adjacent subunit for the WT and mutant proteins). Source data are provided as a Source Data file.

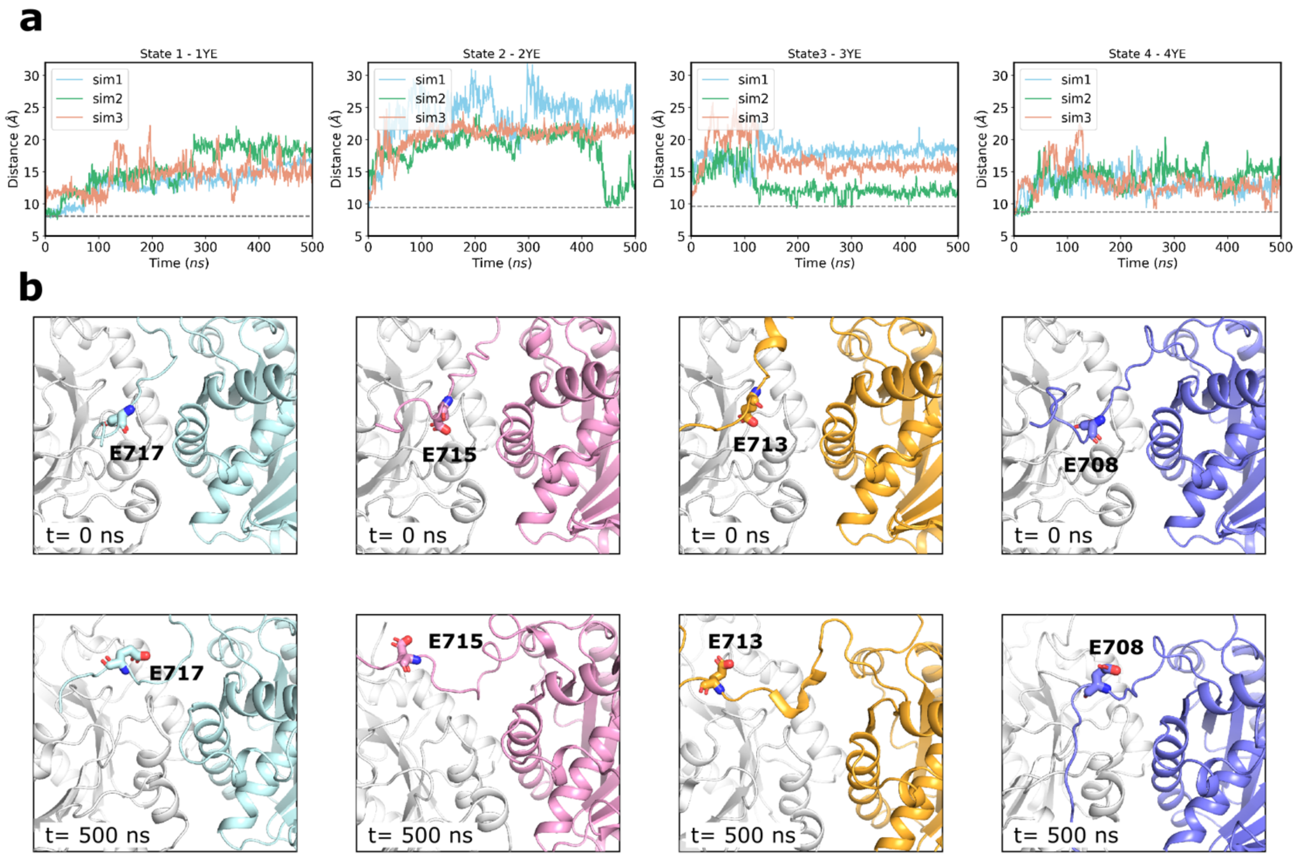

Supplementary Figure 8. Flexibility of the C-terminal tail of the Tyr-to-Glu mutants. a Time traces of the distances between the Cβ of the mutated Glu residue and the Cα of D564 from the adjacent subunit for each state. The dashed lines indicate the distance at $t = 0\ ns$. **b** Comparison between the initial and final frames of each state showing that the Tyr-to-Glu mutations result in the displacement of the Glu residues from the active site. Source data are provided as a Source Data file.

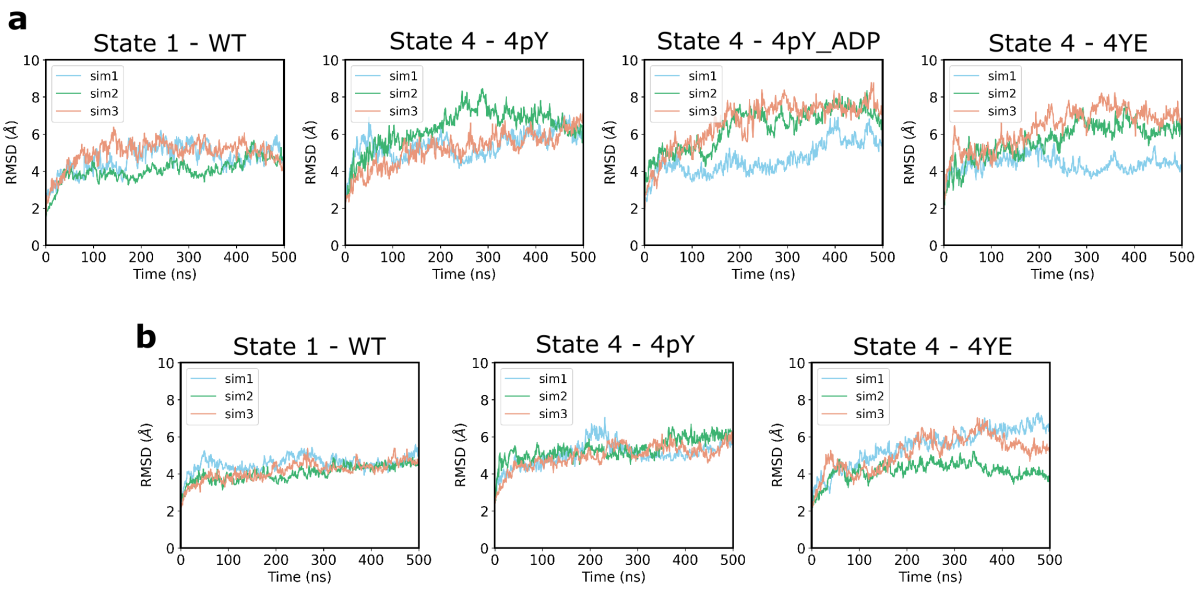

Supplementary Figure 9. RMSD time traces for the dimeric (a) and trimeric (b) proteins. RMSDs were calculated considering the entire dimeric or trimeric structure. Source data are provided as a Source Data file.

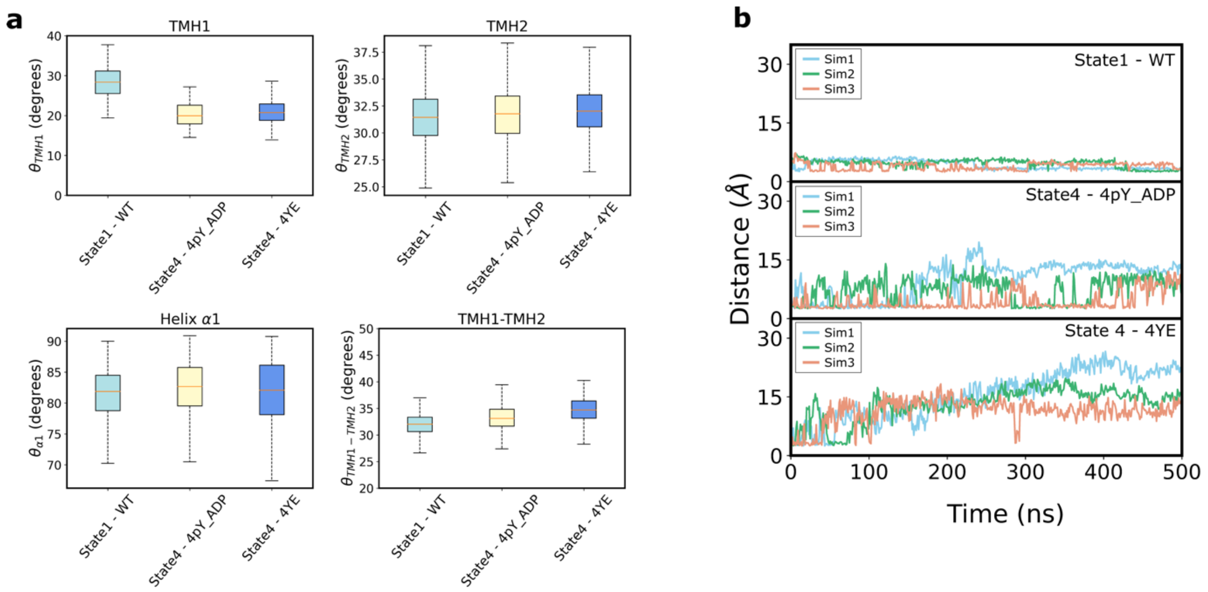

Supplementary Figure 10. Molecular dynamics analyses of the transmembrane signal transduction in trimeric systems. **a** Boxplot of the angles described in Fig.6a (n=1500 MD simulations frames. The box bounds the interquartile range divided by the median, with the whiskers extending to a maximum of 1.5 times the interquartile range beyond the box). **b** Time trace of the minimum distance between the residues D28 and K701. Source data are provided as a Source Data file.

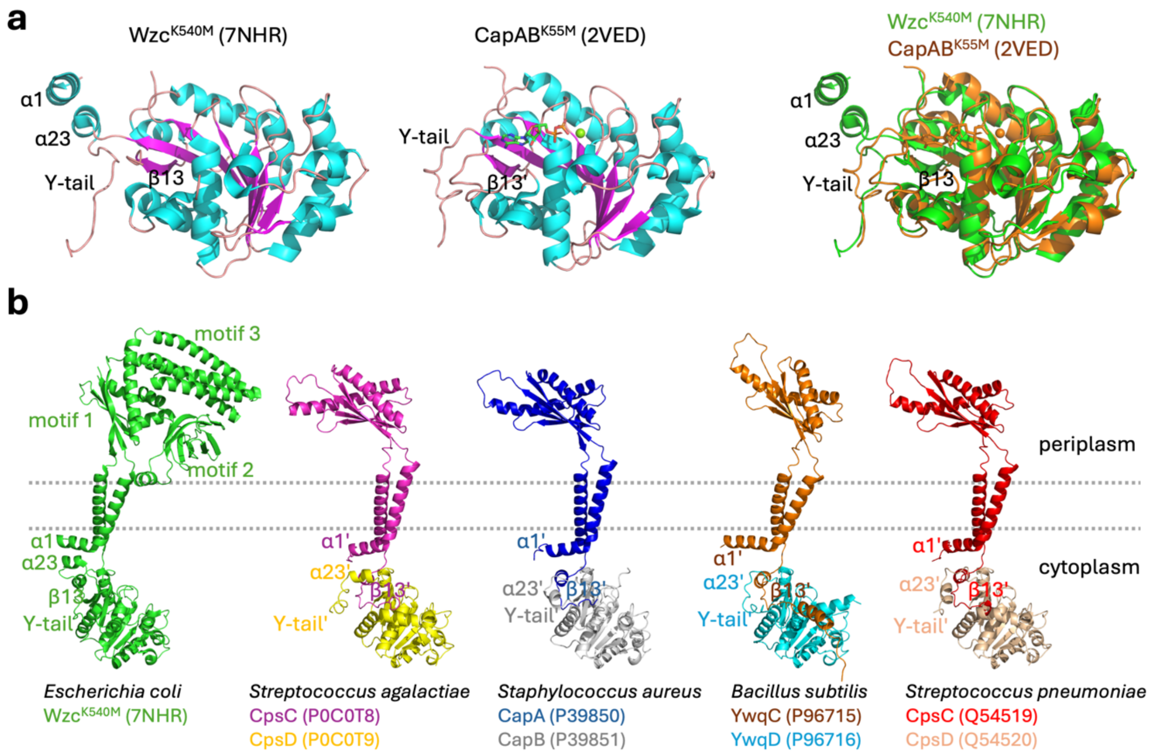

Supplementary Figure 11. Structural comparison of *E. coli* K30 Wzc and homologs from Gram-positive bacteria. a The cytoplasmic region of Wzc^{K540M} cryo-EM structure (PDB ID: 7NHR) (left), the crystal structure of CapAB^{K55M} (PDB ID: 2VED) from *Staphylococcus aureus* (middle), and the comparison of the two (right). The left two structures are coloured according to the secondary structure. **b** Monomeric structure of *E. coli* Wzc^{K540M} and the AlphaFold models of the Gram-positive homologs. The PDB ID of *E. coli* Wzc^{K540M}, and the Uniprot IDs of the Gram-positive proteins are shown. To construct the schematic models of the Gram-positive systems, the AlphaFold models of the transmembrane protein and the cytoplasmic protein were aligned to structure of *E. coli* Wzc^{K540M} (PDB ID: 7NHS). The positions equivalent to α1, α23, β13, and the tyrosine-rich tail (Y-tail) in *E. coli* Wzc^{K540M} are indicated as α1', α23', β13' and Y-tail', respectively. The AlphaFold models used are: AF-P0C0T8-F1-model_v4, AF-P0C0T9-F1-model_v4, AF-P39850-F1-model_v4, AF-P39851-F1-model_v4, AF-P96715-F1-model_v4, AF-P96716-F1-model_v4, AF-Q54519-F1-model_v4, AF-Q54520-F1-model_v4. Note that the AlphaFold models suggest there are structural changes in helix α23 for the Gram-positive homologs.

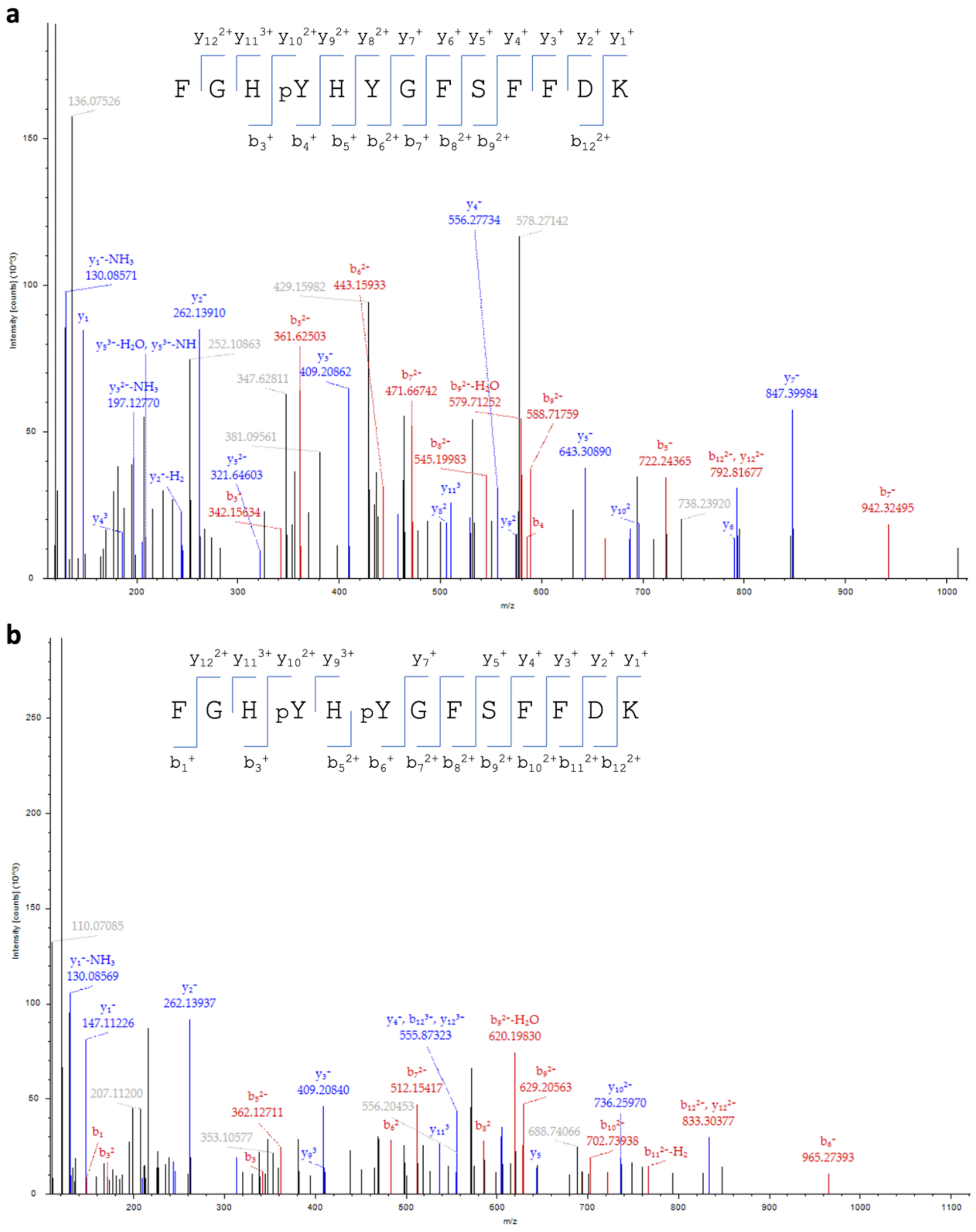

Supplementary Figure 12. MS/MS spectrum of the tryptic phosphopeptide (aa. 708-720) from purified WzcYF_713Y/N711Y. a Fragment ions of isolated precursor ion (m/z 577.9063) showing mono-phosphorylation at N711Y. **b** Fragment ions of isolated precursor ion (m/z 605.2304) showing phosphorylation at both N711Y and Y713.

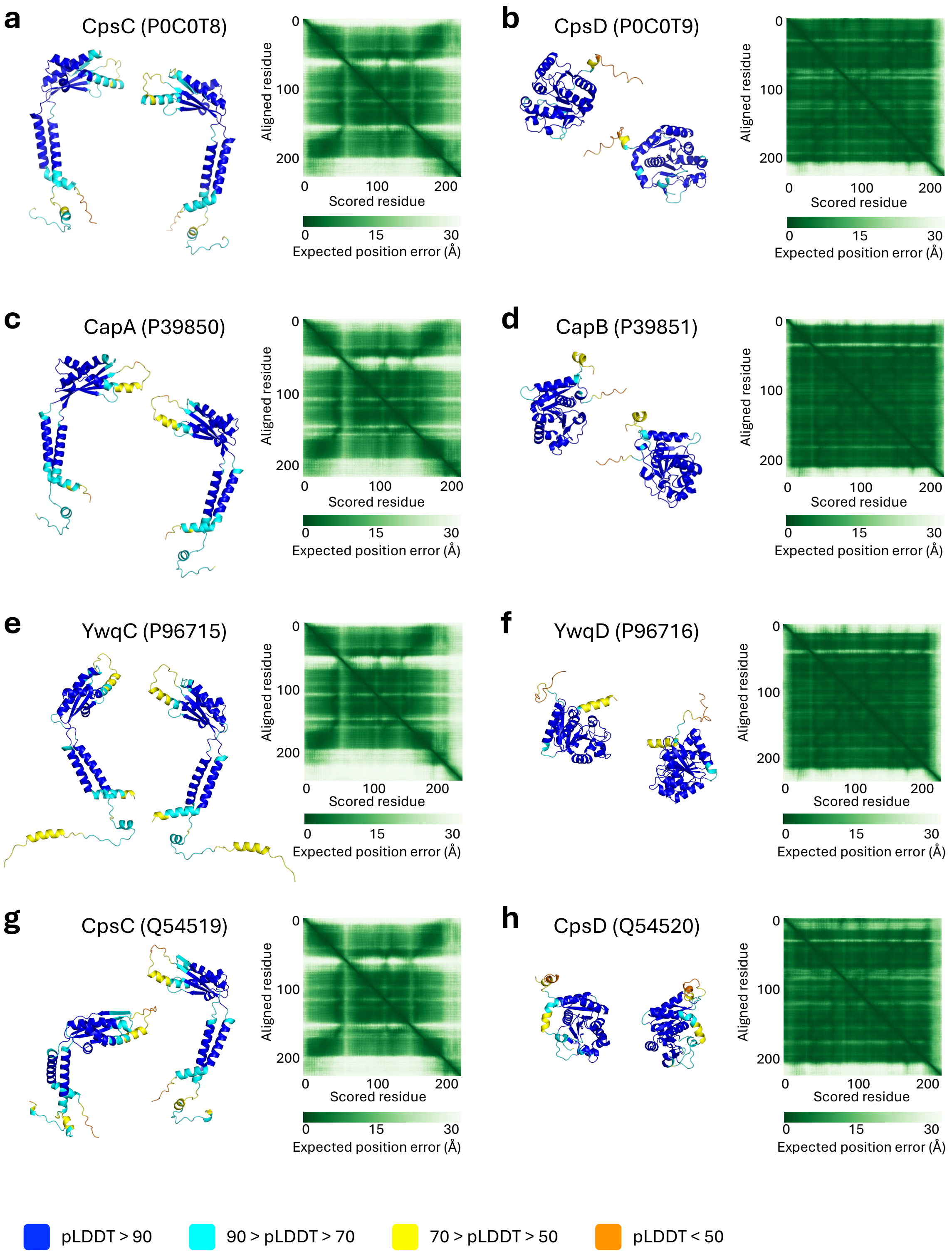

Supplementary Figure 13. The AlphaFold models. The predicted models are shown with the pLDDT score overlaid from two viewing angles (left two of each panel) and the predicted aligned error (PAE) (right of each panel). The Uniprot ID for each model is shown. The color code of pLDDT score is shown at the bottom.

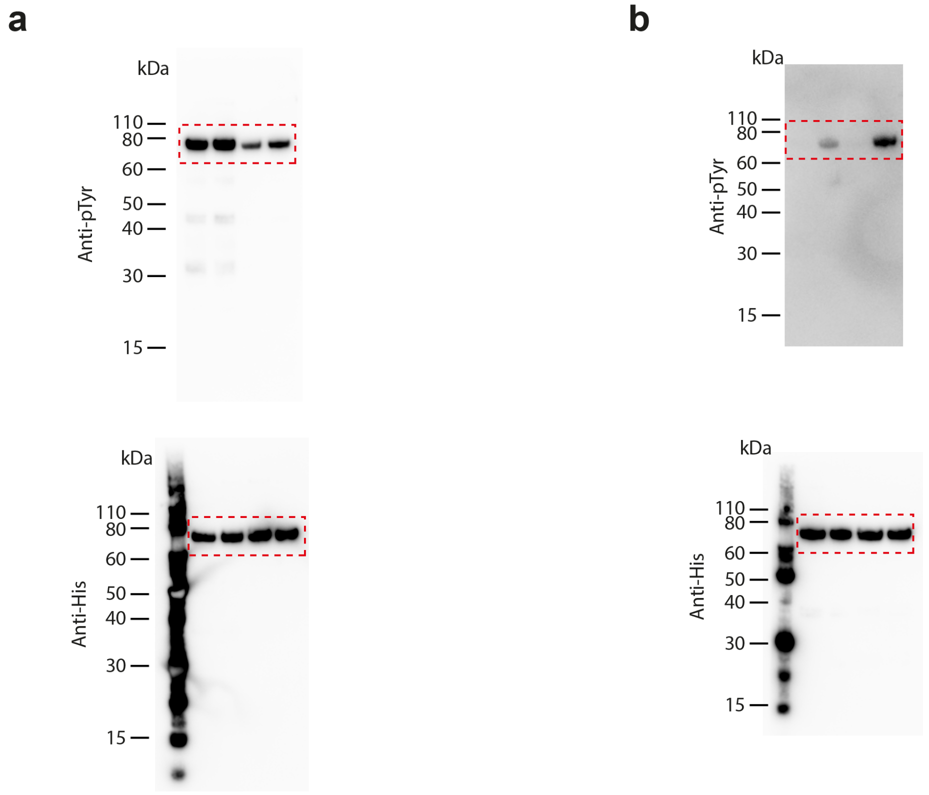

Supplementary Figure 14. Uncropped immunoblots for panels shown in Figure 7b (a) and 7c (b). The antibodies indicated by labels on each panel and the cropped region shown in the figures is identified by the dashed red boxes.
